# Transition to and from the skyrmion lattice phase by electric fields in a magnetoelectric compound

**DOI:** 10.1038/ncomms12669

**Published:** 2016-09-01

**Authors:** Y. Okamura, F. Kagawa, S. Seki, Y. Tokura

**Affiliations:** 1 Department of Applied Physics and Quantum Phase Electronics Center, University of Tokyo, Tokyo 113–8656, Japan; 2 RIKEN Center for Emergent Matter Science (CEMS), Wako 351–0198, Japan; 3 PRESTO, Japan Science and Technology Agency, Bunkyo, Tokyo 113–8656, Japan

## Abstract

Dissipation-less electric control of magnetic state variable is an important target of contemporary spintronics. The non-volatile control of magnetic skyrmions, nanometre-sized spin-swirling objects, with electric fields may exemplify this goal. The skyrmion-hosting magnetoelectric chiral magnet Cu_2_OSeO_3_ provides a unique platform for the implementation of such control; however, the hysteresis that accompanies the first-order transition associated with the skyrmion phase is negligibly narrow in practice. Here we demonstrate another method that functions irrespective of the transition boundary. Combination of magnetic-susceptibility measurements and microwave spectroscopy reveals that although the metastable skyrmion lattice is normally hidden behind a more thermodynamically stable conical phase, it emerges under electric fields and persists down to the lowest temperature. Once created, this metastable skyrmion lattice remains without electric fields, establishing a bistability distinct from the transition hysteresis. This bistability thus enables non-volatile electric-field control of the skyrmion lattice even in temperature/magnetic-field regions far from the transition boundary.

## Abstract (web summary)

Magnetoelectric material Cu_2_OSeO_3_ hosts topologically-protected skyrmion magnetization textures, however only in an impractically narrow temperature range. Here, the authors demonstrate how the metastable skyrmion phase may be extended to low temperatures by an applied electric field.

## Graphical abstract

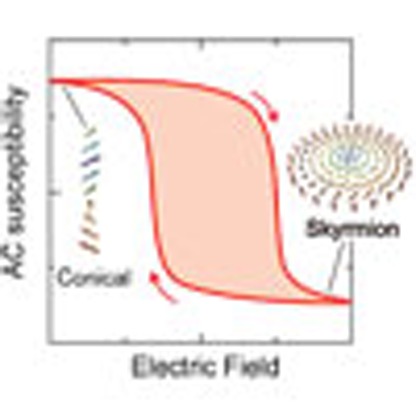

The magnetic skyrmion with an integer topological number is now ubiquitously identified in noncentrosymmetric magnets^1^,^2^,^3^,^4^,^5^,^6^,^7^,^8^,^9^,^10^,^11^, providing versatile controllability depending on the electrical nature of the skyrmion-hosting material^12^,^13^,^14^,^15^,^16^,^17^,^18^,^19^,^20^,^21^. In metallic systems, the coupling of skyrmion lattice to conduction electrons enables, for instance, current-driven translational motion with a low threshold of current density^12^,^13^,^14^ and the stochastic creation/annihilation of individual skyrmions with a spin-polarized current^15^. In insulating systems, skyrmion phase control can be expected to be feasible without Joule-heating dissipation by means of electric fields. In particular, since the discovery of magnetoelectric skyrmions in the insulating material Cu_2_OSeO_3_ (ref. 8), the non-volatile control of skyrmion lattice phase with electric fields has been long sought.

To achieve non-volatile phase control, in general, two requirements must be met. First, the material of interest should possess a bistability of competing binary states in the absence of external stimuli, for example, magnetic and electric fields; we note that the exact degeneracy of the two states is not necessarily required. Second, the more thermodynamically stable state should be interchanged when positive and negative external fields are applied. These two requirements are always satisfied in the hysteresis region of a first-order transition; however, the hysteresis of the transition between the skyrmion phase and another magnetic order (a conical state) is too narrow in width to be observed in the magnetoelectric Cu_2_OSeO_3_, therefore, the first requirement is not fulfilled in practice with regard to this conventional hysteresis. Moreover, no report has described how the thermodynamic stabilities of the skyrmion phase and the competing conical phase change in response to the application of electric fields. Because of these two open issues, the electric field switching of the skyrmion phase in Cu_2_OSeO_3_ has remained as a challenge.

In this work, we explore one other bistability that exists far from the phase boundary. Combination of magnetic-susceptibility measurement and microwave spectroscopy reveals that metastable skyrmion lattice is potentially hidden behind the conical phase and can be created by applying electric fields. Once created, this metastable state remains even without the electric field, establishing bistability even far from the phase boundary. By controlling both thermodynamic stability and metastability, the skyrmion lattice can be created and annihilated with electric fields isothermally, realizing non-volatile switching. This finding can lead to conceptually new guiding principle of non-volatile skyrmion phase control that is applicable to other external stimuli.

## Results

### Thermodynamic stability under electric fields

**Figure 1:**
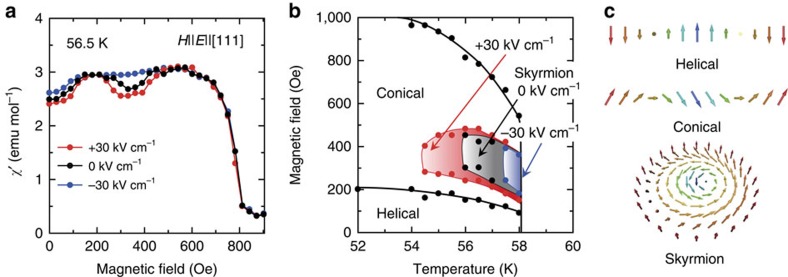
Electric field variation of the thermodynamic stability of the skyrmion phase. (**a**), The magnetic field dependence of the a.c. susceptibility, *χ'*, under various electric fields of −30 (blue), 0 (black), and +30 kV cm^−1^ (red). (**b**), The magnetic phase diagram near the transition temperature under electric fields of −30 (blue), 0 (black), and +30 kV cm^−1^ (red). Sign of electric field is defined as parallel (+) and anti-parallel (−) to the magnetic-field direction. (**c**), Schematics of helical, conical and skyrmion structures. The helical region in the phase diagram comprises multi-domains with different wave vectors (**q**'s) of spin helix, while the conical region represents the single **q**-state helix along the magnetic-field direction.

We first address how the thermodynamic stability of the skyrmion phase varies under electric fields by measuring the a.c. susceptibility *χ′* as a sensitive probe for skyrmion formation^8^,^21^. We chose the magnetic field (*H*) || [111] configuration, where the electric polarization (*P*) appears also along the [111] direction. We thus applied the electric field (*E*) along the [111] direction and the sign of electric field is defined as+and−when *E* is parallel and anti-parallel to *P*, respectively^20^ (Supplementary Fig. 1). Figure 1a summarizes the typical magnetic field profiles of *χ′* under zero/positive/negative electric fields, just below the magnetic transition temperature. Note that the dip anomaly in *χ′*, which is a well-known indicator of the skyrmion phase, is clearly seen at approximately *H*=350 Oe at both *E*=0 and +30 kV cm^−1^, whereas there is no such anomaly at −30 kV cm^−1^; furthermore, this dip appears in a wider range of magnetic fields at +30 kV cm^−1^ than at 0 kV cm^−1^. These behaviours indicate that the thermodynamic stability of the skyrmion phase is enhanced or undermined by the application of positive or negative electric fields, respectively. This tendency is also evident on the temperature axis in the phase diagrams constructed under different electric fields (Fig. 1b) and can be well explained by considering that larger values of electric polarization are observed in the skyrmion phase than in the conical phase^8^,^21^ (see also Supplementary Fig. 1 for the polarization data and the same behaviour is observed in the *H*||[110] and *P*||*E*||[001] configuration (Supplementary Fig. 2)). We note that this observation can be interpreted also in terms of the electric-field-induced change in uniaxial magnetic anisotropy: The driving force of electric-field-induced phase transitions can be expressed by the electrostatic energy gain, −*PE*. In the present material, the *P* originates from the magnetoelectric effect, and according to ref. 22, *P*∝(*m*_[010]_*m*_[001]_, *m*_[001]_*m*_[100]_, *m*_[100]_*m*_[010]_) (we define *m*_[100]_ as the [100] component of the spin and so on). Hence, when the magnetic field is along the [111] direction, *P* is also along [111] and proportional to *m*_[111]_^2^. This conclusion indicates that the electric-field-induced free energy change (*E*||[111]), –*PE*, can be mapped onto the uniaxial anisotropy change (∝–*m*_[111]_^2^*E*), and therefore, *E*>0 (< 0) leads to the easy-axis uniaxial (easy plane) anisotropy. According to the theoretical predictions^23^,^24^ and the recent experiment^25^, the easy-axis uniaxial (easy plane) anisotropy stabilizes (destabilizes) the skyrmion lattice phase, consistent with our observation (Fig. 1b).

### Emergence of metastable skyrmion lattice

**Figure 2:**
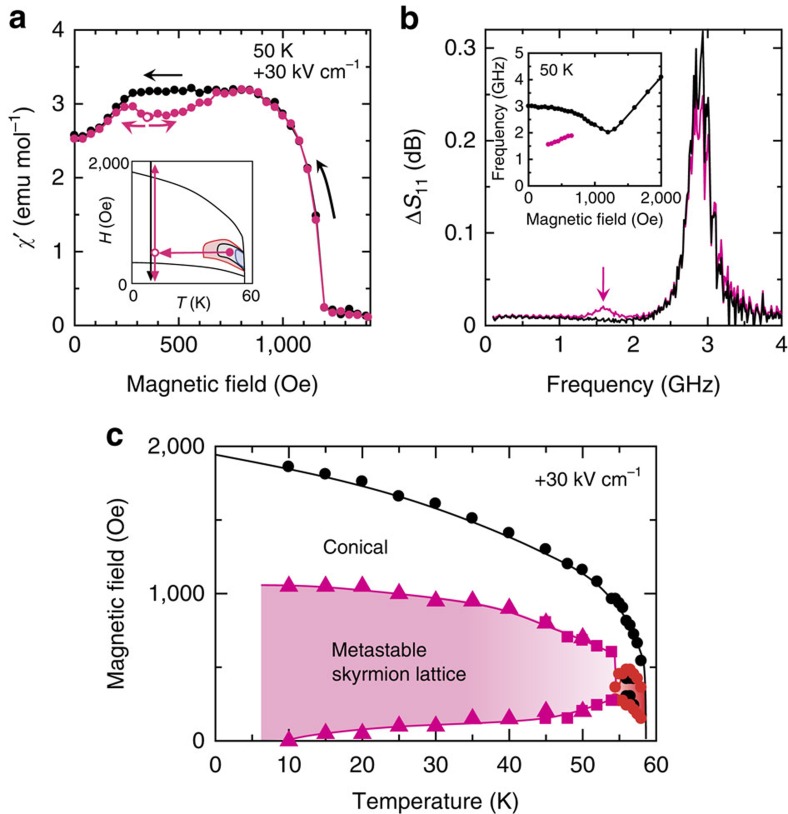
Metastable skyrmion lattice realized by magnetic- and electric-field cooling. (**a**), The magnetic field dependence of the a.c. susceptibility *χ'* measured after magnetic-field (350 Oe) cooling under a field of +30 kV cm^−1^ and then scanning the magnetic field up and down (magenta symbols), and alternatively by decreasing the magnetic field from the ferromagnetic phase (black symbols). The measurement procedures are schematically illustrated in the inset. *T* and *H* represent the temperature and magnetic field, respectively. The magenta and black arrows represent the measurement procedures for the data shown in the main panel with the corresponding colour. The open magenta symbol in the main panel corresponds to the point indicated by the open magenta symbol in the inset. (**b**), The microwave spectra measured after the ME cooling (magenta) and without ME cooling (black). Δ*S*_11_ represents the microwave absorption coefficient. (inset) The magnetic field dependence of the resonance frequencies derived from the microwave spectra measured after the ME cooling. (**c**), The magnetic phase diagram including the metastable skyrmion state, under an electric field of +30 kV cm^−1^. Data points marked by magenta squares and triangles were determined via a.c. susceptibility measurements and microwave spectroscopy, respectively.

The other open issue is the temperature/magnetic field region in which the skyrmion phase and the competing conical phase are bistable. Although the existence of a bistable region other than the conventional hysteresis is generally nontrivial, here we find that metastable skyrmion lattice is hidden deep in the conical phase; namely the bistability can emerge even far from the transition hysteresis. We first show the emergence of the metastable skyrmion lattice by performing magnetic field cooling with the application of a positive electric field; this cooling process is hereafter termed the magnetoelectric (ME) cooling. Figure 2a displays the *χ′*–*H* profiles recorded under a field of +30 kV cm^−1^ at 50 K, which is well below the lower-temperature boundary of the skyrmion phase. A dip anomaly similar to that observed at 56.5 K (Fig. 1a) is observed when the ME cooling is performed to pass through the skyrmion phase, indicating the presence of the metastable skyrmion lattice under a field of +30 kV cm^−1^ even at 50 K. The same conclusion can also be drawn from the results of microwave spectroscopy^26^,^27^; in addition to a higher-frequency absorption peak (∼2.8 GHz), another absorption peak appears at ∼1.6 GHz only when the ME cooling is performed (Fig. 2b). The magnetic-field dependence of these resonant modes is consistent with the literature^26^,^27^ (Fig. 2b inset), therefore, the resonant modes at 2.8 and 1.6 GHz in Fig. 2b can be assigned to the magnetic resonance of the conical spin structure and the skyrmion counterclockwise rotational mode, respectively, thus proving that the skyrmion phase persists at this temperature and coexist with the thermodynamically stable conical phase. Moreover, magnetic field cooling without any electric field results in no dip anomaly (Supplementary Fig. 3), conversely highlighting the vital role of the electric field in accessing the metastability hidden behind the conical phase. This metastable state persists even down to the lowest temperature, leading to marked expansion of the skyrmion-existing region (Fig. 2c), whereas the skyrmion phase is limited to a narrow phase pocket under the absence of an electric field (see also Supplementary Fig. 4).

### Metastability under electric fields

**Figure 3:**
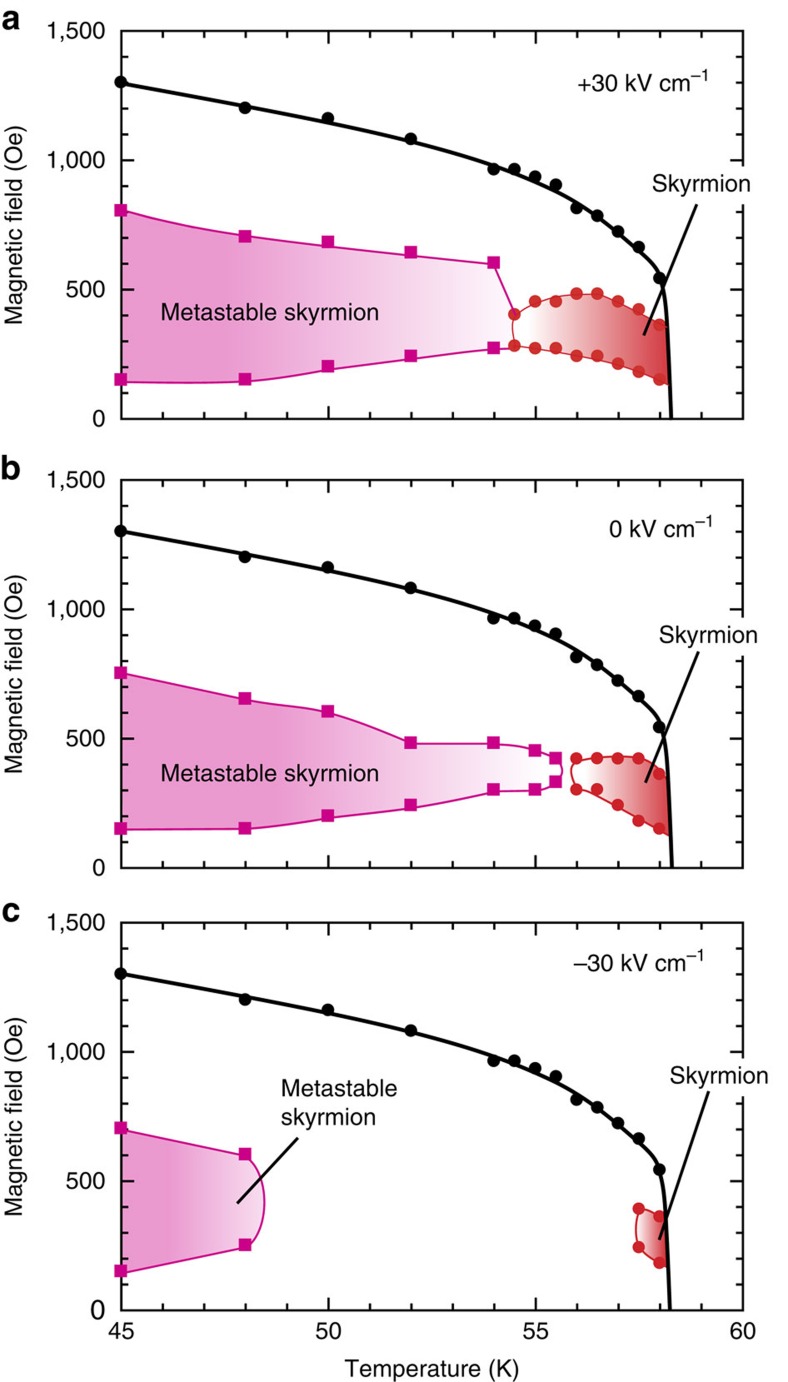
Variation in the thermodynamic stability and metastability of the skyrmion phase under selected electric fields. (**a**–**c**), The high-temperature portion of the magnetic phase diagrams, including the metastable skyrmion states that are created by the ME cooling procedures (*E*=+30 kV cm^−1^, *H*=350 Oe), under electric fields of +30 (**a**), 0 (**b**) and −30 kV cm^−1^ (**c**). Red- and magenta-colored regions represent the thermodynamically stable and metastable skyrmion lattice states, respectively.

Remarkably, at low temperatures, the metastable skyrmion lattice created via the ME cooling process is found to remain after the applied electric field is switched off or even inverted; thus, we can investigate the magnetic field ranges in which metastable skyrmion lattice exists and hence bistability holds at each temperature under various electric fields (for the details of the measurement procedure and the corresponding *χ′*–*H* profiles, see Supplementary Fig. 5). Figure 3a–c show the phase diagrams constructed under various electric fields for the metastable states that are beforehand created by the ME cooling procedures (*E*=+30 kV cm^−1^, *H*=350 Oe). Unlike the conventional bistability or metastability, which adheres to the first-order phase boundary, the metastable skyrmion region spreads to lower temperatures in all phase diagrams. Nevertheless its area strongly depends on the magnitude of the electric field: At 0 kV cm^−1^, for instance, the skyrmion (practically) unstable region exists between the skyrmion phase and the metastable skyrmion region (Fig. 3b), consistent with the fact that the metastable skyrmion lattice is not present after magnetic field cooling without any electric field (Supplementary Fig. 3). At +30 kV cm^−1^, contrastingly, the skyrmion phase and the metastable skyrmion region are connected with each other (Fig. 3a) whereas these regions are distinctly separated at −30 kV cm^−1^ (Fig. 3c). The experimental metastable state is dictated by not only the existence of the metastable solution but also its lifetime^28^. According to refs 2, 23, the solution of the skyrmion lattice, that is, the metastability can persist in a wider (narrower) magnetic-field region under the easy-axis uniaxial (easy plane) anisotropy. In our experiment (Fig. 3), the positive (negative) electric field leads to wider (narrower) metastable regions, consistent with the theoretical predictions, given that the application of positive (negative) *E* introduces the easy-axis uniaxial (easy plane) anisotropy. However, the magnetic-field range of the metastable region is apparently narrower than the predictions, indicating that the lifetime limits the metastable region observed in our experiment.

### Non-volatile switching of skyrmion lattice

**Figure 4:**
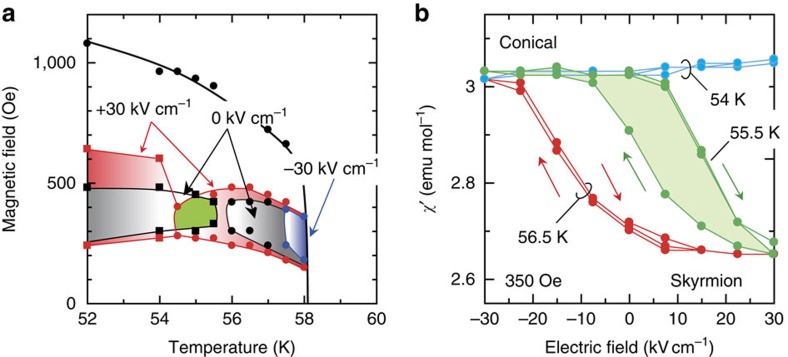
Non-volatile switching between the skyrmion and conical phases using electric fields. (**a**), Superposed phase diagrams measured under different electric fields; +30, 0, −30 kV cm^−1^. The green-hatched region represents the temperature/magnetic field region in which non-volatile switching of the skyrmion phase is possible using electric fields. Red-, black- and blue-coloured regions represent (thermodynamically stable or metastable) skyrmion lattice states under +30, 0 and −30 kV cm^−1^, respectively. The green-hatched region highlights the region where the thermodynamically stable (at +30 kV cm^−1^), metastable (at 0 kV cm^−1^), and unstable (at −30 kV cm^−1^) skyrmion lattice are all realized. (**b**), The *χ'*–*E* profiles at 54, 55.5, and 56.5 K at 350 Oe. The hysteresis loop at 55.5 K is highlighted in green, representing the non-volatile switching between conical (high *χ'*) and skyrmion (low *χ'*) phases.

Up to this point, we have revealed the two necessary conditions for the non-volatile electric switching of the skyrmion lattice; one is the temperature/magnetic field region in which the bistability of the conical phase and the metastable skyrmion lattice holds without an electric field, and the other is the region in which the thermodynamically stable states at +30 and −30 kV cm^−1^ are interchanged between the skyrmion and conical phases. On the basis of these findings, we can now identify the region in which both requirements are satisfied, as highlighted in green in Fig. 4a. Figure 4b displays the *χ′*–*E* profiles measured inside and outside the green-hatched region. At 55.5 K and 350 Oe, a point that is located inside the region of interest, we observe a clear hysteresis in the *χ′*–*E* profile, indicating non-volatile switching between the skyrmion and conical phases (corresponding to smaller and larger *χ′* values, respectively), as expected. We note that such a hysteretic, non-volatile switching of the skyrmion phase is observed only in the green-hatched region, where the thermodynamically stable (at +30 kV cm^−1^), metastable (at 0 kV cm^−1^), and unstable (at −30 kV cm^−1^) skyrmion lattice are all realized. At 54 K and 350 Oe, on the other hand, the skyrmion lattice and the conical spin structure are bistable but they are not interchanged by electric fields of ±30 kV cm^−1^, thus showing no change in the magnetic state with the adopted electric field magnitude. By contrast, at 56.5 K and 350 Oe they can be interchanged but are not bistable; in this case, although the application of the electric field induces a magnetic transition between the skyrmion and conical phases, the switching behaviour is volatile, and the initial magnetic state is recovered when the electric field is turned off.

Even in the green-hatched region, the created skyrmion lattice appear to partially revert to the conical phase when the electric field is released, probably because thermal agitation tends to allow the metastable skyrmion lattice to relax into the thermodynamically stable conical phase. Fully non-volatile switching is therefore expected at lower temperatures; for this, the skyrmion phase must be further extended down to low temperatures, and thus, stronger electric fields are required. Unfortunately, electric fields larger than 30 kV cm^−1^ often lead to the dielectric breakdown of the material used in this study.

## Discussion

We note that the green-hatched region is dictated by both the thermodynamic stability at a given positive electric fields and metastability at zero electric field (Fig. 4a). Whereas, to annihilate the skyrmion lattice, the working point should be located inside the skyrmion unstable region at negative electric fields, the green-hatched region satisfies this condition because the unstable region is widely extended (or, equivalently, the metastable skyrmion region shrinks significantly; Fig. 3c). Notably, the thermodynamically stable phase boundary corresponds to the free energy balancing points of the competing phases, while the metastable phase boundary is dictated by the relaxation, that is, lifetime of the metastable skyrmion lattice^28^. The large electric field variation of the metastable region, therefore, implies that the lifetime of the metastable skyrmion lattice significantly depends on the electric field. Because the conversion from the skyrmion lattice to the conical phase should entail a change in the topological winding number of spins, this process is likely catalysed and limited by the creation of some magnetic singularity^29^,^30^. The electric field dependence of the lifetime may thus be traced back to a unique coupling between such a magnetic singularity and the external electric field, as discussed in recent theoretical studies^31^,^32^.

In conclusion, we have measured magnetic susceptibility and microwave spectra under electric fields to investigate the stability of skyrmion lattice in the magnetoelectric Cu_2_OSeO_3_. The application of electric fields leads to the concomitant change in the thermodynamically stable and metastable skyrmion regions, enabling the non-volatile phase control of skyrmion lattice even far from the skyrmion-conical transition boundary. This guiding principle may also enable the skyrmion switching induced by other external stimuli^25^,^33^.

## Methods

### Crystal growth and sample preparation

Single crystals of Cu_2_OSeO_3_ were grown via chemical vapour transport^8^. For the application of electric fields, we cut the sample with a wire saw and carefully polished it. The typical dimension of the sample was 1.7 × 1.7 × 0.1 mm^3^.

### A.c. susceptibility measurement

We measured the a.c. susceptibility *χ′* using a superconducting quantum interference device magnetometer (Quantum Design, Magnetic Property Measurement System (MPMS)) equipped with a home-built sample holder to enable the application of electric fields. The frequency and excitation amplitude of the a.c. susceptibility measurement are 700 Hz and 3.8 Oe, respectively. The leakage current is less than 1 pA during the measurement at 30 kV cm^−1^. The cooling rate utilized in this study was fixed at 10 K min^−1^. The typical measurement time per measurement point was a few minutes. In the *χ′*–*E* measurement (Fig. 4b), we set a target temperature, then applied sufficiently high magnetic fields to form the ferromagnetic state, and finally reduced the magnetic field to the target point (350 Oe).

### ME cooling

As shown in the inset of Fig. 2a, we chose the skyrmion phase (57 K and 350 Oe) as the starting point and then applied an electric field of +30 kV cm^−1^. Subsequently, we decreased the temperature to a given target point, for instance, 50 K, and finally, the a.c. susceptibility was measured while increasing/decreasing the magnetic field. During all procedures, the electric field was maintained. When moving onto another target temperature, we switched off both magnetic and electric fields after the measurements, then warm up the sample to 57 K and applied a magnetic field of 350 Oe, and finally, after reaching the starting point (57 K, 350 Oe), we applied an electric field and set next target temperature (see also Supplementary Fig. 3).

### Microwave spectroscopy

We performed microwave reflection measurements using a network analyser (E8363C; Agilent Technology) and determined the reflection coefficient *S*_11_ under a magnetic field *H*. The absorption spectrum associated with the magnetic excitations was obtained by calculating Δ*S*_11_ (*H*)=*S*_11_ (3,000 Oe)−*S*_11_ (*H*) because *S*_11_ (3,000 Oe) does not contain the magnetic excitation of interest in the considered frequency window (0.1–5 GHz) and can thus be regarded as the background spectrum. The cooling rate in this experiment was fixed at ∼3 K min^−1^. The difference in the cooling rate between the a.c. susceptibility measurements (∼10 K min^−1^) and the microwave spectroscopy measurements (∼3 K min^−1^) did not affect the results, as shown in Supplementary Fig. 6.

### Data availability

The data that support the findings of this study are available from the corresponding author on request.

## Additional information

**How to cite this article:** Okamura, Y. *et al.* Transition to and from the skyrmion lattice phase by electric fields in a magnetoelectric compound. *Nat. Commun.* 7:12669 doi: 10.1038/ncomms12669 (2016).

## Supplementary Material

Supplementary Information Supplementary Figures 1-6
