# Supplementary material for: Transition to and from the skyrmion lattice phase by electric fields in a magnetoelectric compound
Source: Nat Commun. 2016 Sep 1;7:12669. doi: 10.1038/ncomms12669 (PMC5025781; doi:10.1038/ncomms12669)
Supplement: Supplementary Information — Supplementary Figures 1-6 [file ncomms12669-s1.pdf]

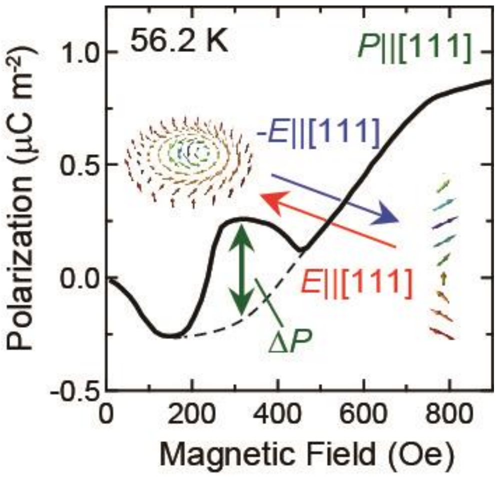

Supplementary Figure 1 | The magnetic field dependence of the electric polarization (*P*). The magnetic-field is applied along the [111] direction, and the electric polarization is measured along the [111] direction. *ΔP* represents the difference between the *P* in the skyrmion phase (solid line) and the expected *P* in the interpolated conical phase (dashed line). Because *P* is larger in the skyrmion phase than in the conical phase, positive and negative electric fields (*E*) are expected to stabilize the skyrmion and conical phases, respectively. Sign of electric field is defined as + and — when *E* is parallel and anti-parallel to *P*, respectively.

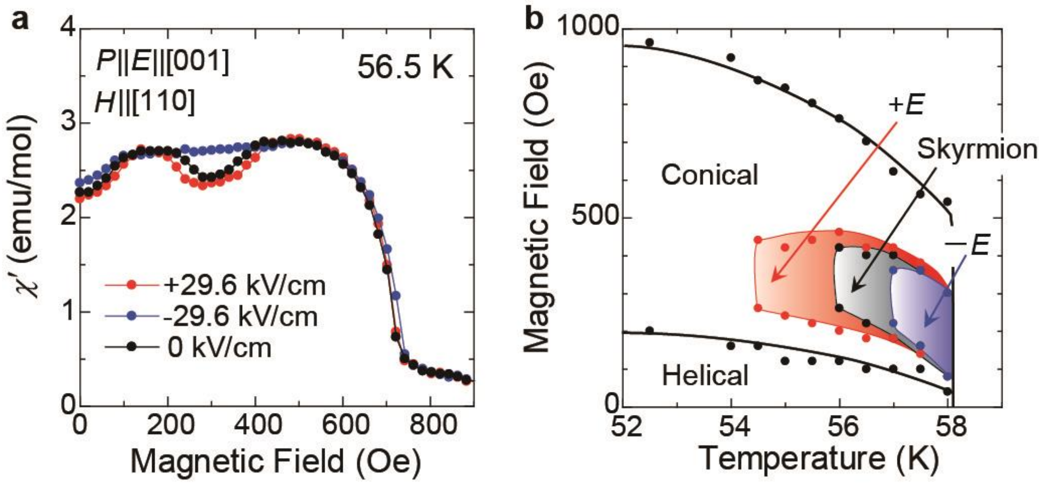

Supplementary Figure 2 | Electric-field-induced change in phase diagram with *H*||[110] and *P*||*E*||[001]. a, The magnetic-field dependence of the a.c. susceptibility, χ', under various electric fields of —29.6 (blue), 0 (black), and +29.6 kV cm^{-1} (red) in the *P*||*E*||[001] and *H*||[110] configuration. **b,** The magnetic phase diagram near the transition temperature under electric fields of —29.6 (blue), 0 (black), and +29.6 kV cm^{-1} (red). Sign of electric field is defined as + and — when *E* is parallel and anti-parallel to *P*, respectively. The thermodynamic stability change in this configuration is almost the same as in the *H*||*P*||*E*||[111] configuration (Fig. 1 in the main text).

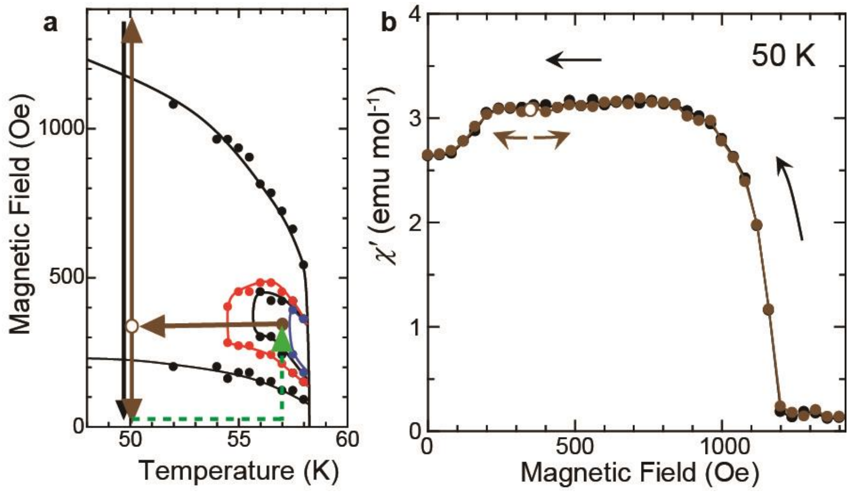

Supplementary Figure 3 | Absence of skyrmions after magnetic field cooling without an electric field. a, The measurement procedures with and without magnetic field cooling under zero electric field, as indicated by brown and black arrows, respectively. When moving onto another target temperature, we switched off magnetic fields after the measurements; then, as indicated by a green dotted arrow, warm up the sample to 57 K and applied a magnetic field of 350 Oe; and finally, after reaching the starting point (57 K, 350 Oe), we set next target temperature. Blue, black and red symbols represent the phase boundary under electric fields of —30, 0 and +30 kV cm^{-1}, respectively. **b,** The magnetic field dependence of the a.c. susceptibility χ' measured after magnetic field cooling (brown) and without magnetic field cooling (black). The open brown symbol corresponds to the point indicated by the open brown symbol in **a**. No difference between the results of the two measurement procedures is discerned within the present experimental accuracy, indicating that the skyrmions induced by magnetic field cooling under a zero electric field do not persist; namely, the metastable skyrmions are readily annihilated while passing through the intervening gap region between the thermodynamically stable and metastable skyrmion states under zero electric field.

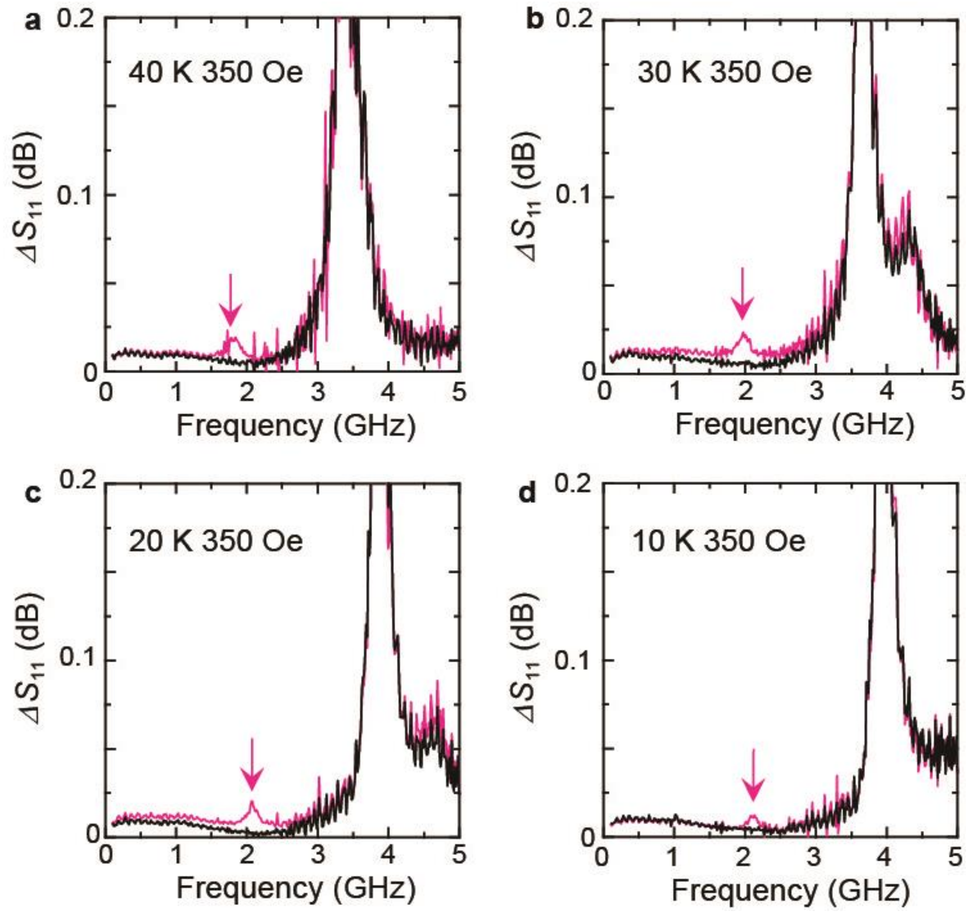

Supplementary Figure 4 | Existence of metastable skyrmions at lower temperatures. **a-d,** The microwave absorption spectra measured after the ME cooling (magenta) and without ME cooling (black) at 40 (**a**), 30 (**b**), 20 (**c**), and 10 K (**d**). The lower-lying peaks, indicated by magenta arrows, originate from the skyrmion counterclockwise rotational mode, proving the existence of metastable skyrmions even at the lowest temperatures.

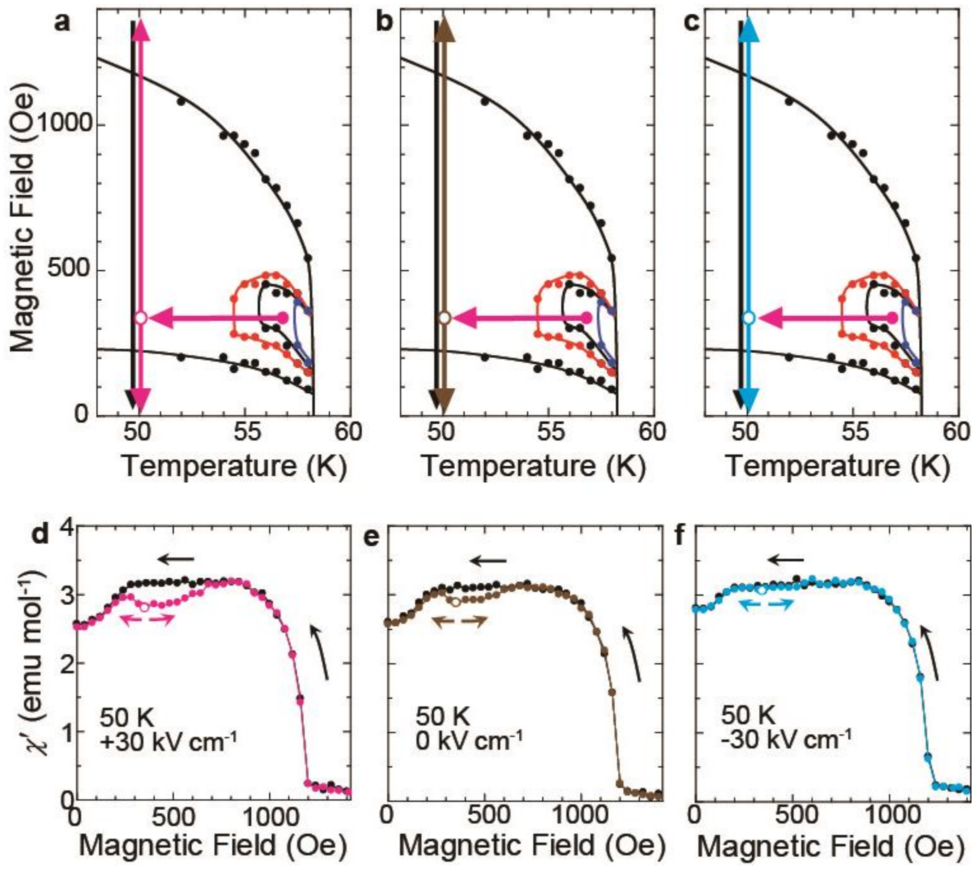

Supplementary Figure 5 | Metastability of the skyrmion phase under selected electric fields as inferred from the a.c. susceptibility measurements. a-c, The measurement procedures for studying the metastability of the skyrmion phase under various electric fields. First, to prepare metastable skyrmions at low temperatures, we applied an electric field of +30 kV cm^{-1} in the skyrmion phase (57 K, 350 Oe) and then lowered the temperature to a given target temperature (for instance, 50 K). Next, we changed the electric field to a target value of +30/0/−30 kV cm^{-1}. Finally, we measured the a.c. susceptibility while increasing/decreasing the magnetic field. Magenta, brown and cyan arrows represent magnetic field- or temperature-scan procedures under electric fields of +30, 0, and −30 kV cm^{-1}, respectively. The black arrows represent the magnetic field-sweep procedures. When moving onto another target temperature, we switched off

both magnetic and electric fields after the measurements; then warm up the sample to 57 K and applied a magnetic field of 350 Oe; and finally, after reaching the starting point (57 K, 350 Oe), we applied an electric field and set next target temperature (see also Supplementary Fig. 3). **d-f,** The magnetic field dependences of the a.c. susceptibility measured using the described procedure.

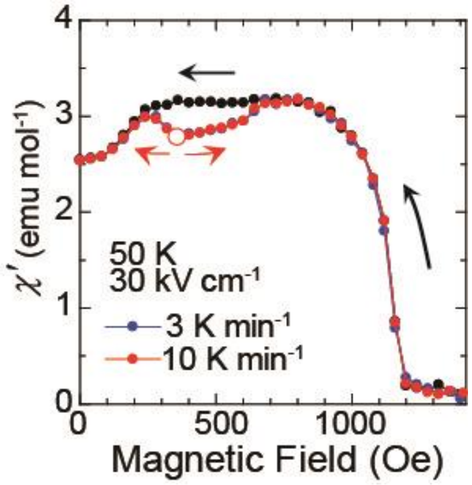

Supplementary Figure 6 | Cooling rate effect on metastable skyrmions. The magnetic field dependence of the a.c. susceptibility χ' measured after the ME cooling at ~ 10 K min^{-1} (red symbols) and ~ 3 K min^{-1} (blue symbols) and measured without the ME cooling (black symbols). Within this cooling rate range, the results are identical.
